# VEXAS syndrome-associated tumefactive demyelination

**DOI:** 10.1007/s00415-025-13239-1

**Published:** 2025-07-12

**Authors:** Tiffany Eatz, Sakir Humayun Gultekin, Namrata Sonia Chandhok, Kristine H. O’Phelan, Sebastian Koch

**Affiliations:** 1 Department of Neurology, The University of Miami, Miller School of Medicine, 1400 NW 12th Ave, Miami, FL 33136 USA; 2 Department of Pathology and Laboratory Medicine, The University of Miami, Miller School of Medicine, 1400 NW 12th Ave, Miami, FL 33136 USA; 3 Department of Hematology, The University of Miami, Miller School of Medicine, 1400 NW 12th Ave, Miami, FL 33136 USA

**Keywords:** VEXAS, New, Novel, Tumefactive demyelination, Seizures, Epilepsy, Syndrome, Differential

## Abstract

**Introduction:**

Vacuoles, E1 enzyme, X-linked (Xp11.3), autoinflammatory, somatic (VEXAS) syndrome is a novel acquired disorder of adulthood, discovered in 2020. Neurological symptoms and sequelae of this new disease are underreported and rarer than their systemic counterparts. We aim to shed light upon the neurological manifestations of this disease by reporting a complex case of a 58-year-old male with a biopsy supporting tumefactive demyelination in the setting of VEXAS syndrome.

**Case Report:**

A 58-year-old male with a history of VEXAS syndrome (diagnosed in 2020 as only myelodysplastic syndrome (MDS)), diabetes mellitus, and relapsing polychondritis presented to our institution’s emergency department with an acute onset of right lower extremity weakness and headache in April 2022. His weakness progressed to right lower extremity hemiparesis with extinction in sensory modality. He was evaluated for acute stroke, with initial differential diagnosis favoring acute venous infarct from cerebral venous thrombosis (CVT) secondary to MDS. However, brain magnetic resonance imaging (MRI) suggested tumefactive demyelination or acute disseminated encephalomyelitis (ADEM) with a left parietal focus, and diagnostic catheter cerebral angiogram found no evidence of CVT. The patient then developed partial status epilepticus without a history of seizures and later became obtunded with global aphasia upon eventual awakening. Subsequent MRI substantiated tumefactive demyelination or glioma. The lesion was biopsied, displaying no neoplastic cells, supporting diagnosis of tumefactive demyelination. The patient received 1 g of daily solumedrol for 5 days and a few months of prednisone taper, with resolution of mental status despite persistence of right-sided hemiplegia and global aphasia. In March 2023, the patient’s genetic testing revealed a *UBA1* gene mutation, solidifying a diagnosis of VEXAS syndrome. At this time, the patient exhibited Broca’s aphasia with intact comprehension. He was neurologically stable in a wheelchair.

**Conclusion:**

To our knowledge, this case is the first reported in the literature of a VEXAS syndrome-associated central demyelination. Further research into the molecular mimicry and pathogenesis of VEXAS syndrome and its neurobiological involvement is strongly encouraged. A growing body of literature will increase comprehension of this novel disease and its role in cerebral pathology.

## Introduction

Vacuoles, E1 enzyme, X-linked (Xp11.3), autoinflammatory, somatic (VEXAS) syndrome is a novel acquired disorder of adulthood, first described by Beck et al. in 2020 [1]. VEXAS syndrome is triggered by a somatic missense mutation in the ubiquitin-like modifier-activating enzyme 1 (*UBA1*) gene that affects hematopoietic progenitor cells and instigates myeloid-mediated autoinflammation [1–4]. This disease process originates in the blood but causes systemic inflammation with multi-organ involvement and associated progressive bone marrow failure [2, 3].

VEXAS syndrome has a late onset, usually after the age of 40 [4]. It is more common in males than females, with a 97.9% predilection for the male sex [4]. Only a few females with acquired X chromosome monosomy or structural deletion have been reportedly afflicted with VEXAS syndrome [4–7].

Signs and symptoms may include fevers, skin rashes and nodules, neutrophilic dermatosis, bone marrow vacuoles, cytopenia (mainly macrocytic anemia with concomitant thrombocytopenia), pulmonary infiltrate (neutrophilic alveolitis, pleural effusions, and bronchial artery vasculitis), chondritis, vasculitis, thrombosis, arthralgias, abdominal pain, diarrhea, scleritis, episcleritis, and iritis, along with neurological phenomena [1, 8]. Clinical manifestations may be categorized as either autoinflammatory or hematological [4], rendering VEXAS syndrome a prototype for a new disease category.

VEXAS syndrome is often fatal and results in significant morbidity and mortality [2–4].

Patients with VEXAS syndrome have been found to respond poorly to immunosuppressive therapies, with the exception of high-dose glucocorticoids [4]. There is currently no optimal protocol for standard of care, and mainstay of treatment remains elusive [2].

We aim to expound upon the neurological manifestations of this disease by reporting a complex case of a 58-year-old male with tumefactive demyelination in the setting of VEXAS syndrome.

## Case Report

A now 58-year-old male with a past medical history of VEXAS syndrome since 2020 (only diagnosed at the time as myelodysplastic syndrome (MDS)), diabetes mellitus, and relapsing polychondritis presented to our institution’s emergency department with an acute onset of right lower extremity weakness and headache in April 2022. His weakness progressed to right lower extremity monoparesis with extinction in sensory modality on neurological examination and right upper extremity pronator drift. Brain computed tomography scan (CT) demonstrated confluent hypodensity in the left parasagittal frontoparietal region and cingulate gyrus, and chest CT showed pulmonary edema and enlarged mediastinal and hilar lymph nodes.

The following day, the patient’s clinical condition rapidly worsened, as he became completely obtunded with left gaze deviation, hemiplegia of the right side, and inability to follow commands despite being conversant less than two hours earlier. There was then a concern for focal seizure in the setting of a hemorrhagic transformation of venous infarct.

**Fig. 1:**
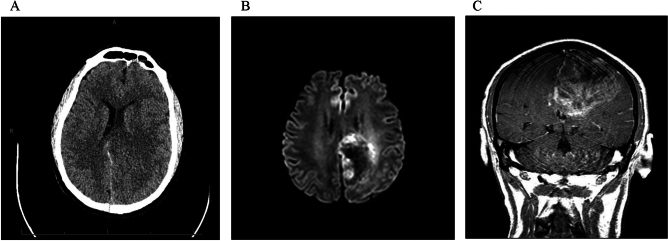
Day 2 brain imaging. **A** Axial CT brain without contrast demonstrating interval worsening of the left paramedian parieto-occipital hypoattenuation with more severe mass effect and rightward midline shift. **B** Axial DWI MRI with plus without contrast demonstrating left parietal lobe lesion with involvement of the splenium of the corpus callosum with a leading edge of diffusion restriction. **C** Coronal MRI post-gadolinium contrast T1-weighted image depicting patchy enhancement involving the left parietal lobe and midline shift

He immediately underwent CT of the brain, which demonstrated interval worsening of the left paramedian parieto-occipital hypoattenuation with a more severe mass effect and rightward midline shift (Fig. 1A). There were blood products overlying the cingulate gyrus and interhemispheric falx, which was concerning for worsening venous infarct. He was given IV levetiracetam, 2500 mg IV load, and three doses of 2 mg IV lorazepam without successful resolution of aforementioned symptoms. He was febrile and started on vancomycin and cefepime. Acyclovir was added empirically to cover for potential viral encephalitis. The working diagnosis at this time was venous infarct from cortical vein thrombosis secondary to MDS (instead, unbeknownst at the time, VEXAS syndrome).

A brain magnetic resonance image (MRI), MRI angiogram (MRA) of the head and neck, and MRI venogram (MRV) of the head were then performed. MRI of the brain demonstrated marked worsening of a volume positive FLAIR hyperintensity involving the left parietal lobe, with involvement of the splenium of the corpus callosum, multiple punctate foci of susceptibility, with patchy enhancement throughout the lesion and a leading edge of diffusion restriction abnormality (Fig. 1B, C). There was also evidence of subarachnoid hemorrhage, greatest along the cingulate gyri, with a thin subdural hematoma along the posterior interhemispheric falx. MRA of the head and neck was unremarkable. Contrast MRV showed loss of signal in the left parietal convexity cortical veins above parenchymal abnormality, although this could have been related to sulcal effacement with or without small cortical venous thrombi. The left transverse sinus was hypoplastic and there was possible low flow in the inferior sagittal sinus. The remainder of the dural venous sinuses were patent. The neuroradiologist’s differential included angioinvasive fungal infection, progressive multifocal leukoencephalopathy immune reconstitution syndrome (PML-IRIS), and acute disseminated encephalomyelitis (ADEM), with other entities such as tumor or tumefactive multiple sclerosis considered less likely.

Diagnostic cerebral catheter angiogram was performed; however, this study was unremarkable and showed no vasculitis. There was no clear evidence of venous sinus thrombosis or cerebral vascular malformation. Cerebral venous thrombosis (CVT) was then removed from the differential. Notably, infectious workup was also negative, and lumbar puncture was not plausible given the patient’s increased intracranial pressure with midline shift and risk for herniation.

**Fig. 2:**
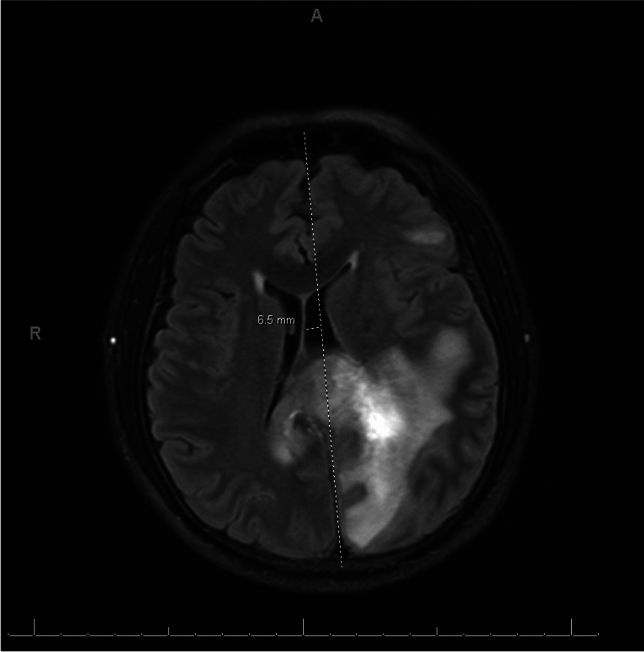
Mid-hospitalization axial MRI brain with plus without contrast displaying evolving hemorrhage within the FLAIR hyperintense enhancing mass-like lesion in the left parietal lobe crossing over the splenium of the corpus callosum with mass effect and midline rightward shift of almost 7 mm

**Fig. 3:**
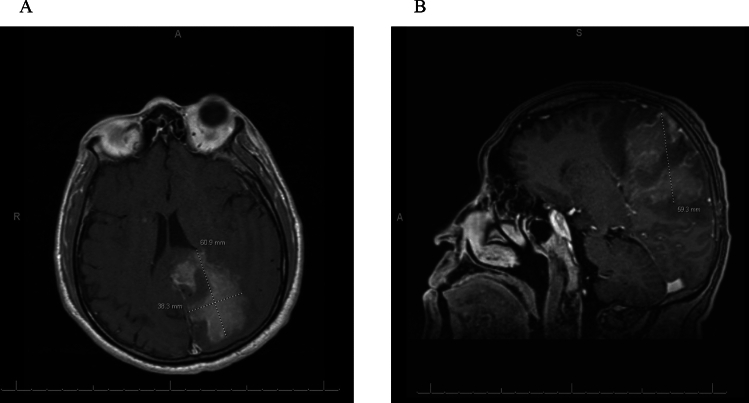
Day 11 brain imaging. **A** Axial T1 MRI with contrast depicting 60.9 × 38.3 mm mass-like lesion, favoring glioma or glioblastoma. **B** Sagittal T1 MP-RAGE with contrast redemonstrating mass-like lesion, favoring glioma or glioblastoma

On day six post-admission, a repeated MRI brain displayed evolving hemorrhage within the FLAIR hyperintense enhancing mass-like lesion in the left parietal lobe that crossed over the splenium of the corpus callosum with mass effect and midline rightward shift of almost 7 mm (Fig. 2). Small foci of bright diffusion-weighted imaging (DWI) signal in the occipital horns of the lateral ventricles were present. This pattern may be seen in infectious ventriculitis, tumor seeding into the lateral ventricles, or hemorrhage extending into the lateral ventricles. Findings were concerning for a high-grade primary brain neoplasm. Differential diagnosis included infectious/inflammatory process and demyelinating disorder, and these MRI findings placed neoplasm high on the differential. Neurosurgery was consulted three days later, and biopsy was carried out four days after that. All the while, the patient remained in similar, stable condition. Interim MRI brain exhibited left uncal herniation and redemonstrated mass-like findings grossly stable compared with the MRI study four days prior (Fig. 3A, B). Glioblastoma or glioma was favored by neuroradiology, with tumefactive demyelination less likely. Lymphoma was also considered less likely because it typically homogeneously enhances and restricts diffusion and is not hemorrhagic. The pattern of signal abnormalities and mass effect is not typical for an infectious process. Tumefactive demyelination and ADEM remained alongside neoplasm on the differential as the most likely etiology of the patient’s condition, and he continued to receive pulse solumedrol 1 g IV for five days followed by oral prednisone 1 mg/kg (60 mg). Throughout this time, the patient remained seizure-free on levetiracetam and slowly clinically improved.

**Fig. 4:**
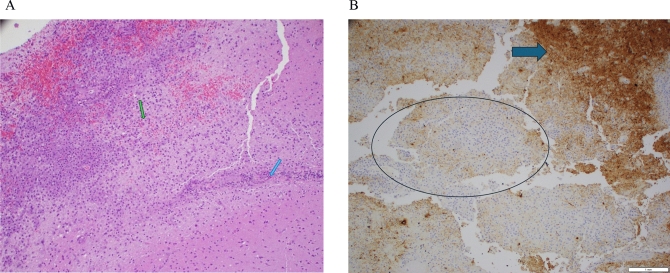
Histopathology. **A** High-power H&E view of the lesion where macrophages (green arrow) become discernible. There are also perivascular lymphocytes on the right (blue arrow). **B** Neurofilament immunostain shows evidence for axonal destruction. The *blue central area (circled)* is devoid of axons as opposed to the peripheral *brown areas* (arrow) where axons are preserved

Brain biopsy histopathology showed a macrophage and T cell-rich process mostly involving subcortical white matter with no obvious primary encephalitis or vasculitis (Fig. 4A). Immunohistochemistry and special stains included LFB for myelin, neurofilament, GFAP, CD68, CD3, SV-40, Congo-Red, GMS, Gram, PAS, HSV-1, Toxoplasma, VZV-1, and CD20. The results were negative for all infectious agents. There were scattered parenchymal and perivascular mature T cells, but no B cells. There was multifocal loss of myelin and some axonal loss (Fig. 4B). There was no perivenular pattern of demyelination. The findings were neither typical for clonal histiocytoses, nor for crystal-storing histiocytosis. CD34 and SMA stains revealed no evidence of vascular wall damage. There was no evidence of amyloid deposits. In general, with the limited tissue samples, an autoimmune or toxic process was difficult to rule out. In retrospect, these findings could be consistent with an autoimmune VEXAS-associated tumefactive demyelination. COVID-associated encephalitis was also considered at the time, given that the patient had COVID in early January 2022.

The patient was discharged to rehabilitation with physical, occupational, and speech therapy 10 days thereafter in stable condition. The patient’s overall hospital stay was 23 days in duration. He was discharged in late May 2022.

In June 2022, the patient came to our neurology clinic for follow-up and had improved speech after rehabilitation therapy. He could not ambulate, and his right-sided hemiparesis and aphasia were improving. There remained some diagnostic uncertainty, although the clinical course was most consistent with tumefactive demyelination. Follow-up brain MRI showed evolution and healing of the left parietal lesion, with a central area of encephalomalacia and no active enhancement. Residual and overall smaller heterogeneous signal intensity lesions in the left, frontal, parietal, and occipital lobes with involvement of the corpus callosum and mild enhancement of the right splenium were seen.

**Fig. 5:**
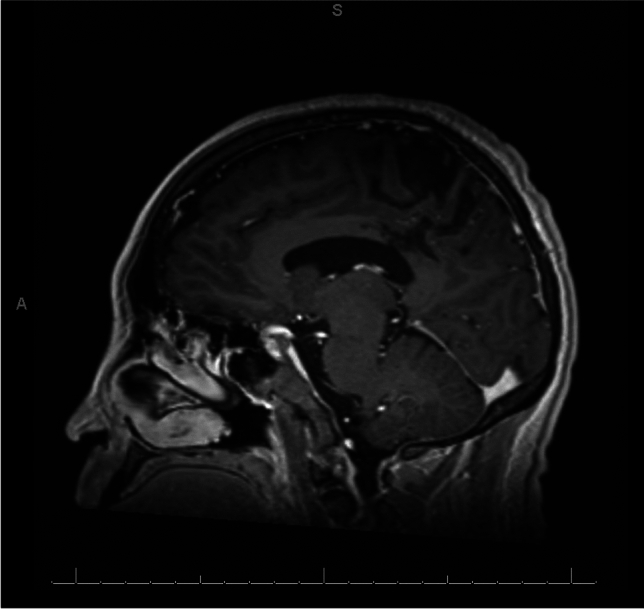
September 2022 sagittal T1 MP-RAGE with contrast demonstrating a healed, treated process of the left parietal lesion with a central area of encephalomalacia and mild enhancement

In September 2022, he was hospitalized for two days for focal status epilepticus that resolved with one dose of 1500 mg of levetiracetam followed by maintenance 1000 mg BID prior to discharge. He presented with right-sided eye, mouth, and upper extremity twitching, which appeared to be automatisms. The patient stated that he was fully aware of the situation but could not express himself due to word finding difficulties during his seizures. His symptomatology was most consistent with temporal lobe partial (focal) aware seizures. EEG showed epileptiform activity arising from the left posterior quadrant. MRI brain during this stay showed no growth of the left hemispheric lesion, with appearances compatible with a treated process (Fig. 5). Mild enhancement may have been related to the loss of the blood–brain barrier in the gliotic center.

In the following months after September 2022, the patient experienced occasional breakthrough seizures on 1000 mg levetiracetam BID. He also struggled with persistent, severe pancytopenia. By March 2023, VEXAS syndrome had become more well known, and our patient received clarity regarding his unrelenting illness since 2020. He underwent genetic testing, which displayed a mutation in the *UBA1* gene at Exon 3, c.122T > C (p.Met41Thr), and was thus diagnosed with VEXAS syndrome. Ruxolitinib was considered as a treatment option. Supportive management and low dose steroids were continued and avatrombopag was considered to assist his thrombocytopenia.

In May 2024, his neurological examination was otherwise stable compared to the previous year. The patient still experienced infrequent breakthrough seizures with the same semiology as previous events, but he has not had any other neurological relapses of his condition.

## Discussion

Our case report details the diagnostic and management challenges presented by the neurological manifestations of VEXAS syndrome. To our knowledge, it is the first reported in the literature of a presumed VEXAS-associated autoimmune brain lesion and central demyelination. In hindsight, we posit that our patient’s tumefactive demyelination and its entailed autoimmunity was associated with his positive VEXAS syndrome status, potentially triggered by molecular mimicry.

Neurological symptoms reportedly attributed to VEXAS syndrome are far less frequent than their systemic counterparts and include hearing loss and cochlea/vestibular dysfunction [4, 9]. Other associated neurological sequelae are more rarely documented in the literature, such as sensory neuropathy, acute attacks of chronic inflammatory demyelinating polyneuropathy, hyperesthesia, proprioceptive anomaly, CVT, and other central nervous system involvement [4, 9–13]. In a recent study investigating the neurological manifestations in VEXAS syndrome patients via the French VEXAS Registry, 17 of 291 patients (6%) had central (30%) or peripheral (70%) nervous system involvement [14]. CNS involvement included six patients with encephalopathy, four with lacunar cerebral infarcts, three with posterior reversible encephalopathy syndrome, and two with optic perineuritis—none of which had tumefactive demyelination [14]. It is our hope that more cases detailing neurological symptoms of VEXAS syndrome are reported as awareness of the syndrome grows.

A germline pathogenic variant in the same *UBA1* gene leads to a syndrome with far higher incidence of severe neurological features than its VEXAS syndrome somatic sister—X-Linked Spinal Muscular Atrophy 2 (SMAX2) [15]. Spinal-bulbar muscular atrophy (SBMA) was also recently discovered to be caused by mutations in this gene [16]. Therefore, the *UBA1* gene plays a considerable role in neurological function, and thus, somatic mutations need to be more comprehensively scrutinized.

It is extremely important to note that our patient’s VEXAS syndrome was not diagnosed until almost a year after his hospital stay. The differential diagnosis throughout the course of our patient’s hospitalization was rapidly changing due to the complexity of the case. Before possible glioma came into play, the competing diagnoses were tumefactive demyelination, ADEM, and CVT, which are immunohematological events with common clinical and MRI features [17]. Evaluation of the course of clinical and radiographic evolution is necessary to differentiate between the three, as was exemplified by our case. Autoinflammation and hematological abnormalities are also the basis of VEXAS syndrome, substantiating the associative pathophysiology of either disorder occurring concurrently [9].

Many of the markers that were radiographically and histopathologically demonstrated in our patient are associated with increased macrophage activity. Tumefactive demyelination lesion histopathology often demonstrates active demyelination with foamy CD68 macrophages, some of which contain myelin, and perivascular lymphocytes [18]. Often axons may be preserved in this disorder [18]. Although our patient’s biopsy did not exhibit myelin-loaded macrophages, it was positive for CD68. Radiographically, our case appears to support tumefactive demyelination over ADEM, because there was only one localized, singular lesion, rather than a disseminated distribution.

Macrophage activation syndrome (MAS) is a reported complication of VEXAS syndrome [19], as it often is in other systemic inflammatory diseases, such as SLE, Sjogren’s disease, and dermatomyositis, among others [20]. MAS criteria are undergoing elaborate standardization via the Delphi International Survey project and consensus [20]. However, the most accepted criteria to date include fever, hepatosplenomegaly, hyperferritinemia, abnormal liver function tests, coagulopathy, thrombocytopenia, hypertriglyceridemia, decreased erythrocyte sedimentation rate (ESR), and bone marrow hemophagocytosis [20]. Our patient exhibited more than half of these symptoms. More literature on cerebral involvement in MAS is required for better understanding. MAS may mimic ADEM or tumefactive demyelination, of which the latter mimics brain neoplasms. However, our patient’s clinical signs and symptoms are still believed to favor diagnosis of tumefactive demyelination rather than MAS [21].

Relapsing polychondritis is also a characteristic of VEXAS syndrome and is comprised of macrophage involvement. Granulation tissue in the perichondrium among normal cartilage tissue in relapsing polychondritis has a high prevalence of CD68+ monocytes and macrophages [22].

Solely speculative in nature, we posit that tumefactive demyelination and VEXAS syndrome may entail macrophagic complicity. Overall, cerebral infiltration of macrophages and microglia in the setting of VEXAS syndrome should be further explored through basic science research.

We encourage further investigation of tumefactive demyelination triggered by VEXAS, potentially through experimental autoimmune encephalomyelitis (EAE) models. Prior EAE mouse models have displayed an increased microglial recruitment and role in demyelination [23]. Tumefactive demyelination EAE can be induced via mouse immunization against MOG with an adjuvant. Identification of specific biomarkers and a translational understanding of microenvironment may be gleaned from such experimentation, as the neurological sequelae of VEXAS syndrome, both microbiologically and macroscopically, are currently undetermined.

### Limitations

The novelty and extreme rarity of VEXAS syndrome restrict case comparison, and there is inherent heterogeneity in medical knowledge and management of this syndrome. To conduct valid and applicable systematic reviews and statistical analyses, more cases of VEXAS need to be encountered and reported in the literature.

## Conclusion

Our case is the first to our knowledge reported in the literature of a presumed VEXAS-associated autoimmune brain lesion, more specifically, tumefactive demyelination. Further research into the molecular mimicry and pathogenesis of VEXAS syndrome and its microbiological neural involvement is strongly encouraged. We also emphasize the need for standardization of care and mainstay treatment protocol of this potentially fatal syndrome. A growing body of literature will increase comprehension of this novel disease and its role in cerebral pathology.
